# On Certain Medical Delusions

**Published:** 1842-12-03

**Authors:** 


### BIBLIOGRAPHICAL NOTICE.

*On certain Medical Delusions. An Introductory Lecture to the Course of Institutes of Medicine in Jefferson Medical College of Philadelphia. Delivered November* 4, 1842. By Robley Dunglison, M. D. Published by the Class. Philadelphia: pp. 34.

This is a sprightly, entertaining, and, in a measure, instructive lecture. Professor Dunglison possesses a happy tact in treating subjects of this character, and on the present occasion has not done himself injustice. At this time when quackery of all kinds is so recklessly encouraged by society, and its promises of speedy success and sure and rapid emolument are so temptingly offered, a sketch of the prominent "medical delusions" which have diverted physicians from the straight paths of science, is an acceptable service to the young student. As a specimen of the Doctor's style and method of treating his subject we subjoin the following paragraphs.

"Every age, gentlemen, has its follies. I have endeavoured to depict some of those that belong to the past, and to our own; and whose decadency we shall witness in no short time, to give place, alas! to others. In the case of homœopathy the revolution has already begun. Hydropathy is supplanting it in Germany, the place of its nativity. In the Homœopathic Hospital at Leipzig, the head quarters of the doctrine, a recent medical traveller found only eight beds; and of these, all but two or three were unoccupied; whilst the village of Gräffenberg was absolutely crowded with those who were undergoing the *Wassercur*, or 'water treatment' of Priessnitz, 'an unlettered and uneducated hind' of the Silesian mountains, who has induced some seven or eight thousand invalids, in the course of the last ten years, to submit themselves for weeks and months to his treatment. Regarding this, there is a growing enthusiasm; and a recent writer—a patient—in his zeal, informs us, that sleeping in wet sheets is by no means the disagreeable thing it is usually conceived to be. The first step may be so; but the subsequent sensations are said to be indescribably delightful.

"There were—we are told—under Priessnitz's care, in 1841, an archduchess, ten princes and princesses, *at least* one hundred counts and barons, military men of all grades, several medical men, professors, advocates, &c., in all about five hundred! 'And besides this high patronage,' adds the same writer, "Priessnitz has accumulated solid pudding to the amount of £50,000; not from the accumulation of guinea fees, or journeys at a guinea a mile, or occasional cheques of £1000 in nightcaps thrown at the surgeon's head, but from fees ranging from the minimum of four shillings a week to the maximum of double that small sum, and from the profit arising from his great boarding house, where his patients are fed for eight shillings a week, and lodged for four shillings more."

"But the reported success of the *Wassercur* is not yet so astounding as that of St. John Long, or of the metallic tractors of Perkins. Amongst those, in England, who furnished vouchers for the value of the tractors as therapeutical agents, with their names affixed to their communications, were eight professors in four different universities, twenty-one regular physicians, nineteen surgeons, thirty clergymen, twelve of whom were doctors of divinity, and numerous other characters of equal respectability; and it was estimated by the London Perkinistic committee, that the number of cures, which had been effected by the tractors up to the period of their report, exceeded *one million five hundred thousand!* And where, it will be asked, is the Perkinistic Institution, where Perkinism itself, now? and Echo answers,—Where? They are both remembered only as the delusions of a by-gone period.

"So goes the world. The Rocks and the Brodums, the Solomons and the Eadys; Perkinism and Thomsonianism, Brandy and Salt, Homœopathy and Hydropathy,

'In turns appear, to make the vulgar stare,
'Till the swoln bubble bursts—and all is air.'

"Happily, one only of those delusions—Thomsonianism—is indigenous with us. The rest are imported. 'Why!' says one of the most distinguished of Irish medical philosophers, in a letter to me, 'why do you not send us something in return for the inflictions of phrenology, mesmerism, homœopathy, &c., which we put upon you?' Yet, Great Britain herself has but adopted these. She derived them, with most of her nursery literature, her Jack and the Bean Stalk, her Jack the Giant Killer, her Tom Thumb, and many of her popular superstitions, from intellectual but imaginative and mystic Germany. Phrenology and mesmerism, homœopathy and hydropathy, are all German; and undoubtedly, in the minds of most, they are more regarded when administered by a German. Yet have they not generally met with honour in their own country;—assuredly with far less than elsewhere.

"First of all, these moral epidemics fade in their primitive seat. Like many well known pestilences, they cross the western main, rage for a while, and ultimately sink beneath the western horizon; to rise again, however, in the east, in the revolution of ages, but under some new phases, and to follow the same path.

"Thus has it likewise been with many popular delusions. Alchemy and the *witch mania* of former ages, are in the 'deep bosom of the ocean buried;' but fortune-telling and astrology yet exist among us.

'And men still grope t' anticipate
The cabinet designs of fate;
Apply to wizards to foresee
What shall and what shall never be.'

"The Mississippi scheme, the South Sea bubble, and the Tulipomania, were the delusions of former periods; but the nineteenth century has been prolific in similar bubbles, and can offer its wild commercial and land speculations; its morus multicaulis mania, and its joint stock companies. The same causes are at the root of all popular delusions. They spring from the credulity of man; his love of the marvellous; his unbounded enthusiasm in the prosecution of whatever may hold out prospects for improving his position, and for ministering to his health and comfort. It is idle, then, to attempt to enact laws against empirical remedies or practices only. To do good, they should be directed against all forms of empiricism and delusion; but even then they must fail. The evil is in the natural constitution of the human mind, and does not admit of eradication. The only feasible course is to educate the people; to instruct them in the operations of the human body; to introduce the study of physiology into the common schools; and to steel the youthful mind, as far as practicable, against the arts of the unprincipled and the ignorant.

"As for the course of the physician it is clear. To avoid even the semblance of persecuting any body of men—however insignificant or unworthy—so as to excite undue sympathy for them. All sects, and the followers of all systems, are glad to raise the cry of persecution; and the people are prepared to believe, that instead of the opposition of the profession being honest and upright, it originates in interested and sordid motives. The observing and reflecting physician can derive information from every sect, and from every form that empiricism assumes, or has assumed. Thomsonianism, homœopathy, and hydropathy have all added to the stock of useful knowledge; and physiology and psychology have been large gainers from phrenology and animal magnetism. Although, therefore, the philanthropist may deplore the pernicious effects of popular delusions on the masses, one consolation remains to him, that science, at least, progresses, and that the cause of truth is ever onward.

"'Let us not, then,'—to conclude, in the language of a very recent writer,—'in the pride of our superior knowledge, turn with contempt from the follies of our predecessors. The study of the errors into which great minds have fallen in the pursuit of truth, can never be uninstructive. As the man looks back to the days of his childhood and his youth, and recalls to his mind the strange notions and false opinions that swayed his actions at that time, that he may wonder at them, so should society, for its edification, look back to the opinions which governed the ages fled. He is but a superficial thinker who would despise and refuse to hear of them, merely because they are absurd. No man is so wise but that he may learn some wisdom from his past errors, either of thought or action; and no society has made such advances as to be capable of no improvement from the retrospect of its past folly and credulity. And not only is such a study instructive: he who reads for amusement only, will find no chapter in the annals of the human mind more amusing than this. It opens out the whole realm of fiction—the wild, the fantastic, and the wonderful, and all the immense variety of things 'that are not, and cannot be; but that have been imagined and believed.'"

